# Supplementary material for: Modeling glioblastoma heterogeneity as a dynamic network of cell states
Source: Mol Syst Biol. 2021 Sep 16;17(9):e10105. doi: 10.15252/msb.202010105 (PMC8444284; doi:10.15252/msb.202010105)
Supplement: Supplementary file 6 — Source Data for Figure 5 [file MSB-17-e10105-s004.zip › Figure5A_sourcedata/GSEA_3071/nosigdb_stateIV.GseaPreranked.1622795164626/OLSEN_MESENCHYMAL.html]

Details for gene set OLSEN\_MESENCHYMAL[GSEA]

|  || Dataset | state53071 |
| Phenotype | NoPhenotypeAvailable |
| Upregulated in class | na\_neg |
| GeneSet | OLSEN\_MESENCHYMAL |
| Enrichment Score (ES) | -0.67072475 |
| Normalized Enrichment Score (NES) | -3.2335427 |
| Nominal p-value | 0.0 |
| FDR q-value | 0.0 |
| FWER p-Value | 0.0 |
Table: GSEA Results Summary

  

Fig 1: Enrichment plot: OLSEN\_MESENCHYMAL      
 Profile of the Running ES Score & Positions of GeneSet Members on the Rank Ordered List

  

| PROBE | GENE SYMBOL | GENE\_TITLE | RANK IN GENE LIST | RANK METRIC SCORE | RUNNING ES | CORE ENRICHMENT || 1 | LHFPL6 |  |  | 52 | 1.815 | 0.0106 | No |
| 2 | LIMA1 |  |  | 58 | 1.805 | 0.0300 | No |
| 3 | COL1A2 |  |  | 137 | 1.425 | 0.0312 | No |
| 4 | ITM2B |  |  | 233 | 1.181 | 0.0264 | No |
| 5 | FOS |  |  | 530 | 0.786 | -0.0214 | No |
| 6 | PRRX1 |  |  | 653 | 0.689 | -0.0370 | No |
| 7 | C1R |  |  | 1072 | 0.502 | -0.1113 | No |
| 8 | EEF1D |  |  | 1660 | 0.357 | -0.2196 | No |
| 9 | RPL32 |  |  | 1887 | 0.315 | -0.2593 | No |
| 10 | B2M |  |  | 2349 | -0.252 | -0.3447 | No |
| 11 | TPT1 |  |  | 2649 | -0.290 | -0.3987 | No |
| 12 | IFITM2 |  |  | 3000 | -0.332 | -0.4619 | No |
| 13 | S100A13 |  |  | 3219 | -0.363 | -0.4996 | No |
| 14 | CCDC80 |  |  | 3492 | -0.408 | -0.5470 | No |
| 15 | LGALS1 |  |  | 3692 | -0.443 | -0.5801 | No |
| 16 | CD99 |  |  | 3752 | -0.456 | -0.5862 | No |
| 17 | CALD1 |  |  | 3763 | -0.458 | -0.5830 | No |
| 18 | PLAC9 |  |  | 4137 | -0.551 | -0.6482 | No |
| 19 | NUPR1 |  |  | 4156 | -0.556 | -0.6453 | No |
| 20 | VIM |  |  | 4255 | -0.582 | -0.6575 | No |
| 21 | IGFBP5 |  |  | 4280 | -0.592 | -0.6554 | No |
| 22 | IGFBP7 |  |  | 4331 | -0.607 | -0.6581 | No |
| 23 | NPC2 |  |  | 4334 | -0.608 | -0.6516 | No |
| 24 | COL14A1 |  |  | 4344 | -0.612 | -0.6464 | No |
| 25 | FTL |  |  | 4350 | -0.615 | -0.6404 | No |
| 26 | FSTL1 |  |  | 4446 | -0.653 | -0.6512 | No |
| 27 | VCAN |  |  | 4464 | -0.661 | -0.6470 | No |
| 28 | PPIC |  |  | 4546 | -0.703 | -0.6545 | No |
| 29 | CST3 |  |  | 4577 | -0.723 | -0.6521 | No |
| 30 | THY1 |  |  | 4587 | -0.729 | -0.6456 | No |
| 31 | RRBP1 |  |  | 4672 | -0.779 | -0.6529 | No |
| 32 | ID3 |  |  | 4686 | -0.788 | -0.6464 | No |
| 33 | IGFBP6 |  |  | 4693 | -0.790 | -0.6387 | No |
| 34 | MGP |  |  | 4824 | -0.890 | -0.6535 | No |
| 35 | SERPINH1 |  |  | 4829 | -0.895 | -0.6441 | No |
| 36 | ZFP36L1 |  |  | 4969 | -1.040 | -0.6590 | Yes |
| 37 | EMP1 |  |  | 4977 | -1.056 | -0.6484 | Yes |
| 38 | UACA |  |  | 5013 | -1.113 | -0.6425 | Yes |
| 39 | NNMT |  |  | 5039 | -1.168 | -0.6341 | Yes |
| 40 | CAVIN1 |  |  | 5050 | -1.185 | -0.6226 | Yes |
| 41 | MT2A |  |  | 5061 | -1.216 | -0.6107 | Yes |
| 42 | TIMP1 |  |  | 5086 | -1.257 | -0.6011 | Yes |
| 43 | COL5A2 |  |  | 5097 | -1.282 | -0.5885 | Yes |
| 44 | GADD45B |  |  | 5099 | -1.292 | -0.5741 | Yes |
| 45 | S100A11 |  |  | 5100 | -1.292 | -0.5595 | Yes |
| 46 | LMNA |  |  | 5116 | -1.325 | -0.5474 | Yes |
| 47 | SERPINF1 |  |  | 5148 | -1.414 | -0.5373 | Yes |
| 48 | S100A6 |  |  | 5158 | -1.485 | -0.5223 | Yes |
| 49 | BGN |  |  | 5160 | -1.486 | -0.5057 | Yes |
| 50 | PRSS23 |  |  | 5179 | -1.562 | -0.4914 | Yes |
| 51 | IGFBP4 |  |  | 5211 | -1.701 | -0.4781 | Yes |
| 52 | COL4A2 |  |  | 5235 | -1.917 | -0.4609 | Yes |
| 53 | DCN |  |  | 5237 | -1.928 | -0.4393 | Yes |
| 54 | TPM2 |  |  | 5242 | -1.971 | -0.4177 | Yes |
| 55 | PCOLCE |  |  | 5247 | -2.050 | -0.3953 | Yes |
| 56 | COL6A2 |  |  | 5258 | -2.256 | -0.3717 | Yes |
| 57 | CTHRC1 |  |  | 5265 | -2.365 | -0.3461 | Yes |
| 58 | COL4A1 |  |  | 5270 | -2.478 | -0.3189 | Yes |
| 59 | LUM |  |  | 5271 | -2.487 | -0.2908 | Yes |
| 60 | MYL9 |  |  | 5272 | -2.509 | -0.2624 | Yes |
| 61 | COL5A1 |  |  | 5276 | -2.605 | -0.2335 | Yes |
| 62 | COL6A1 |  |  | 5278 | -2.689 | -0.2033 | Yes |
| 63 | FN1 |  |  | 5284 | -3.017 | -0.1701 | Yes |
| 64 | TAGLN |  |  | 5285 | -3.021 | -0.1360 | Yes |
| 65 | COL3A1 |  |  | 5287 | -3.508 | -0.0965 | Yes |
| 66 | COL6A3 |  |  | 5288 | -3.654 | -0.0552 | Yes |
| 67 | COL1A1 |  |  | 5290 | -4.897 | -0.0000 | Yes |
Table: GSEA details [plain text format]

  

Fig 2: OLSEN\_MESENCHYMAL: Random ES distribution      
 Gene set null distribution of ES for **OLSEN\_MESENCHYMAL**
